# Novel context-specific genome-scale modelling explores the potential of triacylglycerol production by *Chlamydomonas reinhardtii*

**DOI:** 10.1186/s12934-022-02004-y

**Published:** 2023-01-17

**Authors:** Haoyang Yao, Sanjeev Dahal, Laurence Yang

**Affiliations:** Department of Chemical Engineering, Queen’s University, 19 Division St, Kingston, K7L 2N9 Canada

**Keywords:** Genome-scale modelling, *C. reinhardtii*, Metabolic engineering, Optimization, Systems biology

## Abstract

Gene expression data of cell cultures is commonly measured in biological and medical studies to understand cellular decision-making in various conditions. Metabolism, affected but not solely determined by the expression, is much more difficult to measure experimentally. Finding a reliable method to predict cell metabolism for expression data will greatly benefit metabolic engineering. We have developed a novel pipeline, OVERLAY, that can explore cellular fluxomics from expression data using only a high-quality genome-scale metabolic model. This is done through two main steps: first, construct a protein-constrained metabolic model (PC-model) by integrating protein and enzyme information into the metabolic model (M-model). Secondly, overlay the expression data onto the PC-model using a novel two-step nonconvex and convex optimization formulation, resulting in a context-specific PC-model with optionally calibrated rate constants. The resulting model computes proteomes and intracellular flux states that are consistent with the measured transcriptomes. Therefore, it provides detailed cellular insights that are difficult to glean individually from the omic data or M-model alone. We apply the OVERLAY to interpret triacylglycerol (TAG) overproduction by *Chlamydomonas reinhardtii*, using time-course RNA-Seq data. We show that OVERLAY can compute *C. reinhardtii* metabolism under nitrogen deprivation and metabolic shifts after an acetate boost. OVERLAY can also suggest possible ‘bottleneck’ proteins that need to be overexpressed to increase the TAG accumulation rate, as well as discuss other TAG-overproduction strategies.

## Background

Microalgae have long been a promising class of organism as a synthetic biological chassis due to their high growth rate, efficient photosystem, and simplicity in cultivation. Because of its carbon fixation capability, it is also believed that algal products have less carbon dioxide emissions and are more sustainable in large-scale production [1]. Within all microalgae, *Chlamydomonas reinhardtii* is the best-studied organism in terms of genome annotation and molecular mechanisms, making it the reference organism for studying algal lipid metabolism [2]. Numerous molecular tools for *C. reinhardtii*, including its chloroplast, have also been developed by the research community, making its production of recombinant proteins easier than any other algae [3, 4]. As a result, *C. reinhardtii* has been utilized to make a wide variety of chemicals in the lab and industry. On the high value-added end, non-native proteins are expressed and produced in *C. reinhardtii* as pharmaceutics such as vaccines, antibiotics, and nutritional supplements [5]. This has been the more economically profitable and fruitful direction, and some standard workflows and toolkits have been established [6]. On the other hand, efforts are made to produce biofuels such as biodiesel, biohydrogen, and bio-alcohol from algae, which are chemicals closely related to the primary metabolism [7]. Some remarkable progress is made that increases *C. reinhardtii* lipid and starch contents by up to 2.5-fold by relatively simple modifications [1]. For example, Rengel et al. showed that overexpressing the acetyl-coenzyme-A (acetyl-CoA) synthetase gene can achieve up to 2.4-fold triacylglycerol (TAG) than the control group [8]. Some sophisticated studies have achieved an 8-fold hydrocarbon increment from the controlled *C. reinhardtii*, using gene knock-out, heterologous expression, and triparental conjugation technique [9]. Specifically, Yunus et al. boosted fatty acid conversion to fatty alkane and fatty alkene by introducing enzymes such as FAP and UndA/B [9]. They also increased system fatty acid levels by deleting gene *aas*, thus preventing fatty acid consumption for cellular acyl-ACP replenishment [9]. Moreover, the metabolic response of *C. reinhardtii* to various medium conditions, such as nitrogen deprivation, acetate concentration, or light intensity, is a practical topic to increase TAG production further. Bogaert et al. showed that all biomass components including fatty acids increase in concentration per cell in response to supplementation with high acetate concentration [10].

Despite these findings, algal starch derivatives, lipid derivatives, and hydrogen are not yet economically feasible substitutes for fossil fuels on the market. Most of the experimental studies focus on modifying a few genes or adding a few chemical species into the medium without dramatically changing the cell from the wild type. It is a missed opportunity, as optimizing these new strains or potentially applying them in conjunctions can achieve a much higher yield with minor added costs. The optimization usually requires quantitative measurements from the phenotype, such as RNA-sequencing, proteomic data, and extracellular metabolomics, which are available from many existing studies. Developing an in-silico workflow would greatly assist researchers in systematically understanding the cellular metabolism from phenotype measurements, which is critical to optimize current biofuel-producing strategies and suggesting novel gene targets.

Genome-scale modelling (GEM) is an in-silico tool to systematically simulate cellular expression and metabolism, which is now widely used in biotechnology and infectious disease research. GEM is a species-specific biological reaction network, usually reconstructed by researchers from an annotated genome of the organism. Genome-scale metabolic model (M-model), the most basic yet accessible GEM that focuses exclusively on predicting metabolic fluxes, is the metabolic sub-network with equilibrium constraint applied. M-model is a mathematical linear optimization problem (LP) (Methods, Eqs. (1)–(3)) and can usually be solved within 0.1 seconds using flux balance analysis (FBA) in COBRA Toolbox [11]. Noticeably, FBA is an algorithm that finds extreme flux values within the feasible metabolic range, and it is not explicitly designed for integrating measured expression data, which is referred to as context-specific modelling. In principle, context-specific modelling has better utilities in metabolic engineering than generic M-model, due to the former being constrained directly by omic data to resemble in vivo conditions. Existing algorithms for context-specific modelling are centred mainly around two approaches: limiting the flux of reactions with lowly expressed genes (i.e., GIMME), and defining and supporting a set of core reactions with highly expressed genes (i.e., mCADRE) [12–14]. Most of these existing methods require user-specified parameters such as expression thresholds, making them less objective and less accessible to a wider community. More importantly, the qualitative ‘highly/lowly/not expressed’ criteria is likely too coarse for investigating TAG production, as the target flux is inherently low. Consequently, it is harder for researchers to study insights from the modelling by these algorithms, as well as suggesting metabolic engineering strategies.

**Fig. 1:**
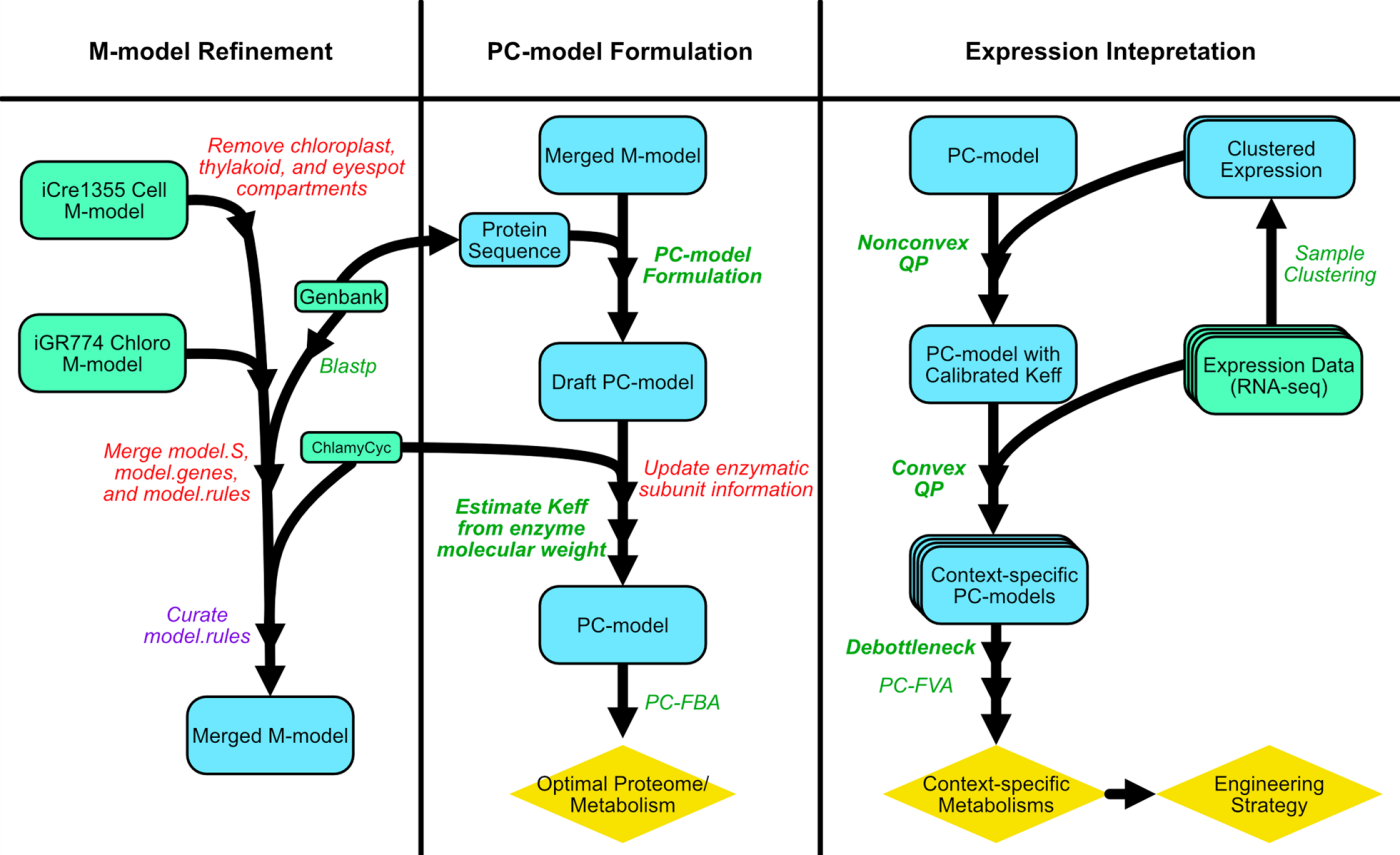
Schematic of the OVERLAY computational pipeline. Boxed texts are files and data, where green rectangle, blue rectangle, and yellow diamond denote starting materials, intermediate steps, and results, respectively. Red italic texts refer to manual procedures, and purple italic texts refer to semi-manual steps with some script aids. In contrast, automatic procedures that are done by the only script are shown in green italic, and methods uniquely developed in OVERLAY are in bold green font

In this study, we develop a computational pipeline, OVERLAY to better address these challenges. We first formulated a protein-constrained metabolic model (PC-model) starting from the published *C. reinhardtii* M-model iCre1355 and chloroplast specific M-model iGR774 [15, 16]. On top of metabolism, PC-model has protein and enzyme concentrations as variables and can be solved using the FBA algorithm with additional benefits. We formulated protein constraint similar to Yurkovich et al., by adding protein concentrations and enzyme concentrations as variables into the M-model, which constrains respective metabolic fluxes [17]. Moreover, expression data from other studies were overlaid onto the PC-model for novel context-specific modelling, which can predict the respective metabolic state using FBA and flux variability analysis (FVA). The workflow of OVERLAY is demonstrated by Fig. 1, which consists of multiple automated algorithms. This will be especially helpful for optimizing bulk material productions from *C. reinhardtii*, and the TAG accumulation case study is done using RNA-seq data from other studies to show the efficacy of OVERLAY.

## Results

### Refined PC-FBA reveals optimal chloroplast metabolism, cellular metabolism, and transportation

We consider *C. reinhardtii* PC-model to be a superior version of the basic M-model, as it can be used for simple analyses such as FBA with better accuracy and more utilities. The total proteome budget has defaulted to a constant of 150 mg per gram of dry cell weight (gDW) (see "Methods" for detailed explanations). A noticeable advantage of PC-model is that exchange reaction boundaries do not need to be set manually. This allows accurate phenotype simulation without uptake flux measurements, thus further offering a convincing comparison of flux networks between different metabolic modes. For example, by assuming sufficient lighting, the photon exchange lower bound can be opened to $-1000$ mmol/gDW/h for any metabolic mode, and the exchange fluxes are solved by the respective optimization. Only the mixotrophic acetate uptake lower bound is manually constrained to $-2$ mmol/gDW/h to mimic a limited substrate availability.

**Fig. 2:**
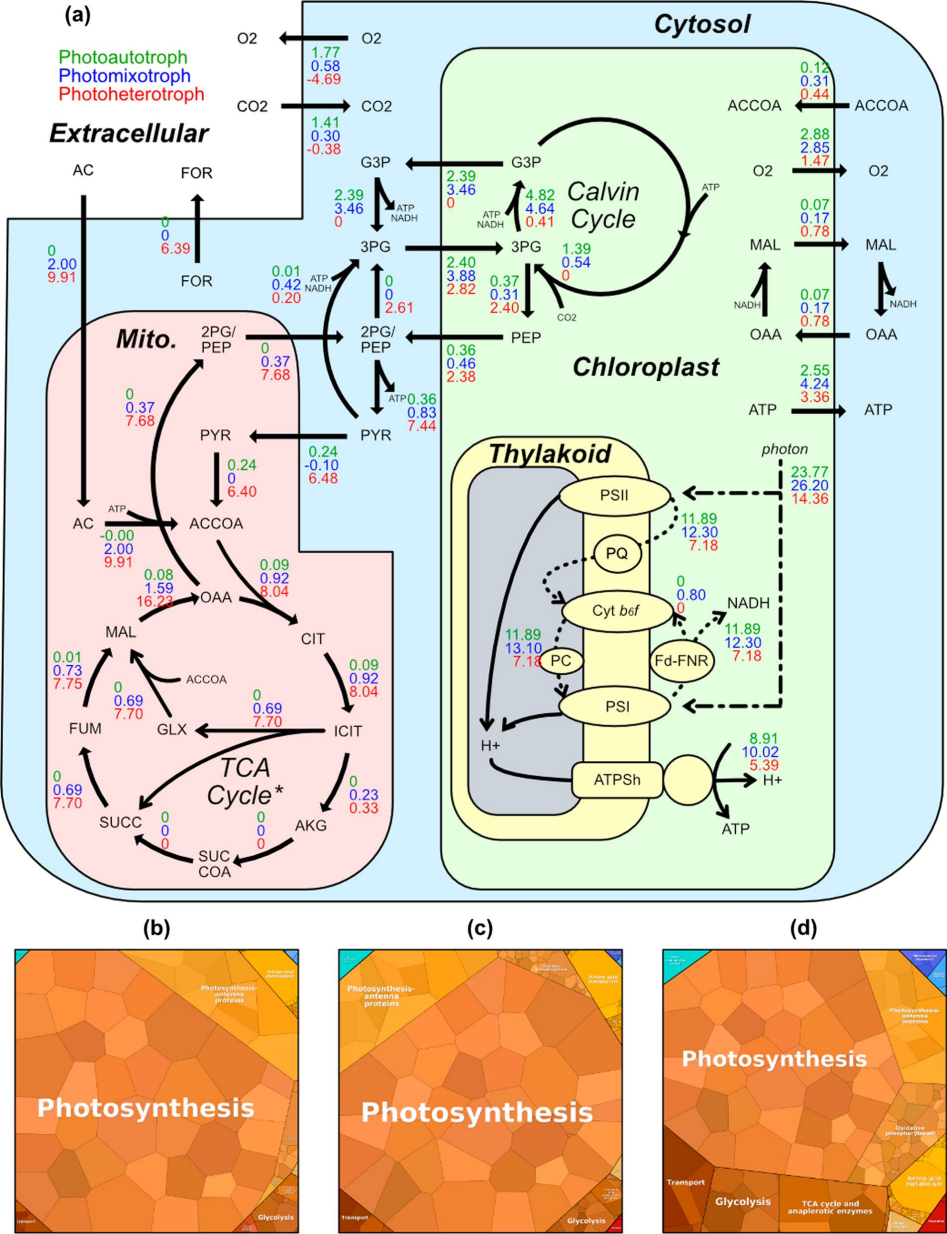
PC-FBA simulation results of autotrophic, mixotrophic, and heterotrophic *C. reinhardtii* growth mode. The optimal metabolic fluxes are shown in **a**, where autotrophic, mixotrophic, and heterotrophic fluxes are denoted by green, blue, and red numbers, respectively. All fluxes are shown in mmol/gDW/h. A negative flux value means the flux is flowing in the opposite direction of the arrow. The dotted lines show the electron flux. All metabolites are shown in BiGG ID. Complex/enzyme abbreviations in thylakoid: PSII, photosystem II; PQ, plastoquinone/plastoquinol; Cyt b6f, cytochrome b6f complex; PC, plastocyanin; PSI, photosystem I; Fd, ferredoxin; FNR, ferredoxin NADP+ reductase; and ATPSh, CF0F1 ATP synthase. The optimal proteome of three growth modes is shown by proteome maps of **b**, **c**, and **d**. Each small polytope is a single protein, and its area denotes the relative abundance. Likely coloured polytope is classified under the same subsystem, which is written in white text. Only modelled proteins are drawn. *Some reactions of the TCA cycle are outside of mitochondria

We used PC-model to simulate the optimal growth strategy in autotrophic, mixotrophic and heterotrophic conditions. We compute metabolic shifts between these conditions focusing on chloroplast metabolism and transportation and interactions with the mitochondria (Fig. 2a). FBA of PC-model (PC-FBA) enabled us to compute the optimal flux-corresponding proteome allocation, of which proteome maps are generated under each growth condition (Fig. 2b–d) [18]. Photosynthesis-associated proteins accounted for 43.2–80.2% of proteome mass in all three conditions (Fig. 2b–d). Correspondingly, photosynthesis largely drives shifts in overall flux states (Fig. 2a).

Our model qualitatively reproduced major and subtle shifts determining the optimal electron flow through photosystems I and II in different conditions. *C. reinhardtii* thylakoid can choose between circular electron flow (CEF) of photosystem I only and linear electron flow (LEF) through both photosystem II and photosystem I, while LEF is the more energy-efficient option [19]. Consistent with this knowledge, the autotroph and heterotroph utilized LEF exclusively to maximize efficiency, as seen by zero flux from Fd to Cyt-b6f (the circular step of CEF) (Fig. 2a). Meanwhile, the mixotroph utilizes CEF, diverting 6% (0.80 mmol/gDW/h out of 13.10 mmol/gDW/h) of electron flux away from NADH/NADPH production back into the CEF. This optimal flux state suggests that CEF can result in faster growth over pure LEF under certain conditions. The result may explain how CEF and LEF are used to balance ATP/reducing power ratio for carbon fixation, as reported by Chaux et al. [20].

Another observation is that, assuming optimal growth, the mixotroph has a higher photosystem activity than the other growth modes: 13.10 mmol/gDW/h electron flux from Cyt-b6f to PC for mixotroph compared to 11.89 and 7.18 mmol/gDW/h for autotroph and heterotroph, respectively (Fig. 2a). These differences are explained by PC-FBA. The autotroph needs a large portion of the proteome budget to conduct other anabolism, such as carbon fixation and gluconeogenesis. These anabolic processes are highly proteome inefficient in comparison with directly consuming organic carbon substrate, rendering autotroph with limited proteome budget for photosystem complexes. For the heterotroph, it is more optimal to spend the proteome budget on consuming acetate, which serves as both an organic carbon source and an energy source. The mixotroph has limited acetate that might be enough for organic carbon to not be forced to run the inefficient anabolism, but insufficient as an energy source, thus having the most potent photosystem to harvest energy. We note that these simulations represent growth-optimized metabolic states. FBA and PC-FBA accurately predict metabolism of adaptively evolved phenotypes [21–23]; however, without additional data-driven constraints these simulations may not resemble wild-type behavior. Indeed, chloroplast content is generally observed to be lower in mixotrophic than autotrophic conditions [24, 25]. Therefore, additional biological mechanisms that are outside the PC-FBA model scope, such as regulation and photoinhibition, may play a significant role in mixotrophic metabolism.

PC-FBA also offers insightful chloroplast metabolism and transport simulations, especially regarding carbon fixation and triose-phosphate transport. As verified by other studies, 9 moles of ATP and 6 moles of NADH are required to produce 1 mole of triose-phosphate from carbon dioxide through the Calvin cycle [26]. Due to this high energy consumption, carbon fixation appears to be a suboptimal growth strategy compared to acetate uptake and is only active when the acetate supply is insufficient.

Under heterotrophic growth, the chloroplast is a net consumer of organic carbon, which is transported as 3-phosphoglycerate. Noticeably, the chloroplast in all phototrophic modes uptakes 3-phosphoglycerate while excreting other triose-phosphate (Fig. 2a). Being the energy supplier of the cell, autotrophic and mixotrophic chloroplast mainly excretes the energy-compact glyceraldehyde-3-phosphate, which has been a phenomenon reported by other studies [26]. The mixotroph has the most active chloroplast, exporting more ATP and reducing power in the form of glyceraldehyde-3-phosphate and oxaloacetate/malate exchange. In all conditions, the chloroplast also consumes various amino acids while producing lipid precursors and six-carbon sugars, which are not shown in the figure in detail.

Our PC-model provides valid mitochondrion fluxomic and cellular exchange simulations for all growth modes. The mixotroph and heterotroph simulations used the glyoxylate shunt in the mitochondria (Fig. 2a), as reported previously by Johnson et al. [26]. This process generates excess oxaloacetate for heterotroph, which is converted to phosphoenolpyruvate and exported to the cytosol from the mitochondria. Additionally, the heterotroph excretes formate, which is only present when proteome constraints are applied (see Additional file 6: Table S1). Thus, the proteome constraints are required to correctly predict respiro-fermentation, or overflow metabolism, as observed in multiple organisms including *C. reinhardtii* [27, 28]. In particular, the optimal heterotroph allocates 43.2% of proteome mass to photosynthesis (Fig. 2d), compared to 80.2% in the mixotroph (Fig. 2c). The reduced photosynthesis protein budget in the heterotroph is allocated instead partially toward glycolysis and TCA cycle proteins (total 15.0%) (Fig. 2d).

Meanwhile, the autotroph shows several contrasting metabolic activities to the heterotroph. The autotrophic mitochondrion has very little activity, mostly powered by importing pyruvate from the cytosol. Instead, it generates phosphoenolpyruvate in the chloroplast, which is transported to the mitochondrion (Fig. 2a). The autotroph does share some characteristics with the mixotroph, such as consuming carbon dioxide and producing oxygen. These PC-FBA simulations suggest that the mixotrophic flux network is more well-balanced and might be the ideal candidate for metabolic engineering.

### Investigating lipid accumulation in nitrogen-deprived *C. reinhardtii* by OVERLAY

Based on insights gained from PC-FBA simulations, we further investigated mixotrophic conditions for bulk metabolite overproduction. The accumulation of TAG, a useful and value-added industrial compound, has been studied extensively in *C. reinhardtii* by inducing nitrogen deprivation. In particular, Goodenough et al. investigated a *sta6* (unable to form starch) strain of *C. reinhardtii*, which showed enhanced TAG accumulation under nitrogen deprivation with acetate boosting 48 h later [29]. The study collected time-course RNA-Seq over four days of culture and discovered highly complex gene expression dynamics: 425 genes up-regulated and over 850 genes down-regulated in response to acetate [29]. Here, we use our OVERLAY to decipher how these complex gene expression dynamics drive flux changes that ultimately lead to enhanced TAG production.

#### OVERLAY Constructs context-specific models with calibrated rate constants

**Fig. 3:**
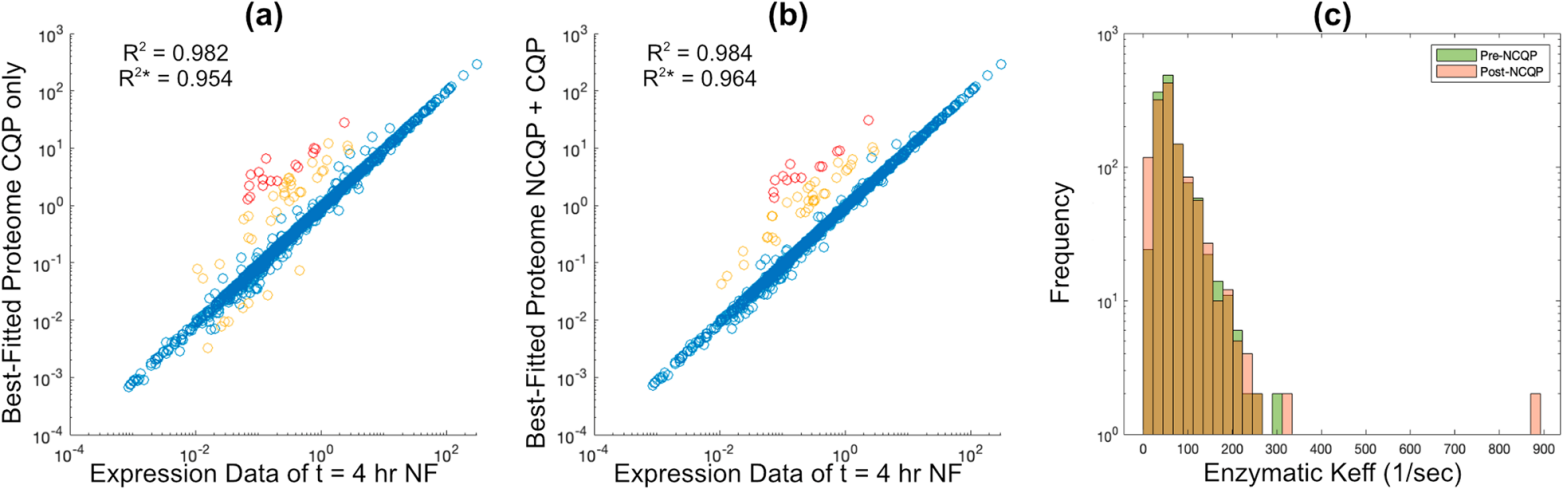
Consistency of simulated proteomes to transcriptomics, and estimated rate constants by OVERLAY. Best-fitted proteome versus measured transcriptomes at t = 4 h before (**a**) and after (**b**) the enzymatic rate constant adjustment by nonconvex QP. $R^{2*}$ is computed using log-transformed data for simulated proteomes and measured transcriptomes, whereas $R^2$ is computed without log-transforming. Outliers are denoted in yellow ($\ge 3$ times) and red ($\ge 10$ times). $R^2$ values are computed using all points, including outliers. **c** Demonstrates $K_\text {eff}$ values before calibration using OVERLAY (pre-NCQP) and after calibration (post-NCQP). Here, $K_\text {eff} = r\cdot K_\text {eff}^\text {avg}$

We first used convex QP only to fit each of the 16 time-course samples onto the PC-model, resulting in 16 context-specific PC-models. Noticeably, enzymatic rate constant ($K_\text {eff}$) values are difficult to determine because most values are not experimentally available. We assume initially that $K_\text {eff}$ are centred around a basal value of $K_\text {eff}^\text {avg} = 65s^{-1}$, and they are proportionally scaled to the SASA [30, 31] (Methods, Nonconvex QP). Across all samples, the best-fitted proteome vectors are consistent with RNA-seq data, with $R^2$ ranging between 0.950 and 0.963, with a median $R^{2*} = 0.958$ (see Additional file 1: Fig. S1a for the complete plot). For example, sample 4 (time = 4 h) has $R^{2*} = 0.954$, with 56 outliers ($\ge 3$ times inconsistency) and 16 far outliers ($\ge 10$ times inconsistency) out of 1495 proteins (Fig. 3a).

Our OVERLAY optimally tuned *r*, or equivalently all $K_\text {eff}$, to achieve the best fit of simulated proteomes to the RNA-Seq, subject to carefully formulated constraints (see "Methods"). We clustered 16 RNA-seq samples into four groups (Additional file 2: Fig. S2), which were used to estimate a single $K_\text {eff}$ vector representing all samples. This results in an improved best-fitted proteome from the original with a median $R^{2*} = 0.966$ across 16 samples (Additional file 1: Fig. S1b). Fig. 3b has 48 outliers, 15 far outliers, and a higher $R^{2*}$ than using convex QP only. Noticeably, the fitting improvement is achieved by varying $K_\text {eff}$ only slightly from $K_\text {eff}^\text {ori}$. According to Fig. 3c, the distributions of $K_\text {eff}$ before and after nonconvex QP adjustment are similar, although a few enzymes are assigned much higher $K_\text {eff}$ than before. Only 214 out of 1222 enzymes have a $K_\text {eff}$ different from $K_\text {eff}^{ori}$ due to extra constraints on OVERLAY (Methods, Eq. (19)), which are placed to reduce the number of total adjustments to $K_\text {eff}$.

Additionally, OVERLAY helps to quality control the metabolic reconstruction, especially regarding its gene-reaction association. We identified a set of proteins whose abundances could not match measurements across all samples. Because we allowed for adjusted rate constants, we hypothesized that the reason for these inconsistencies is due to mis-annotations in the original model reconstruction. We manually inspected all 15 proteins that are far outliers in at least 8 out of 16 samples and compared them with their annotated functions in ChlamyCyc and ALGAEPATH (see Additional file 6: Table S2a for the full list and details) [32, 33]. Indeed, we found that 5/15 proteins had incorrect gene–protein-reaction associations in the reconstruction (see Additional file 6: Table S2a). Of the remaining ten inconsistent proteins, we found potential isozymes for four proteins. We found a total of eight potential isozymes (Additional file 6: Table S2b), which are promising candidates for future studies.

#### Context-specific PC-models providing new metabolic insights

**Fig. 4:**
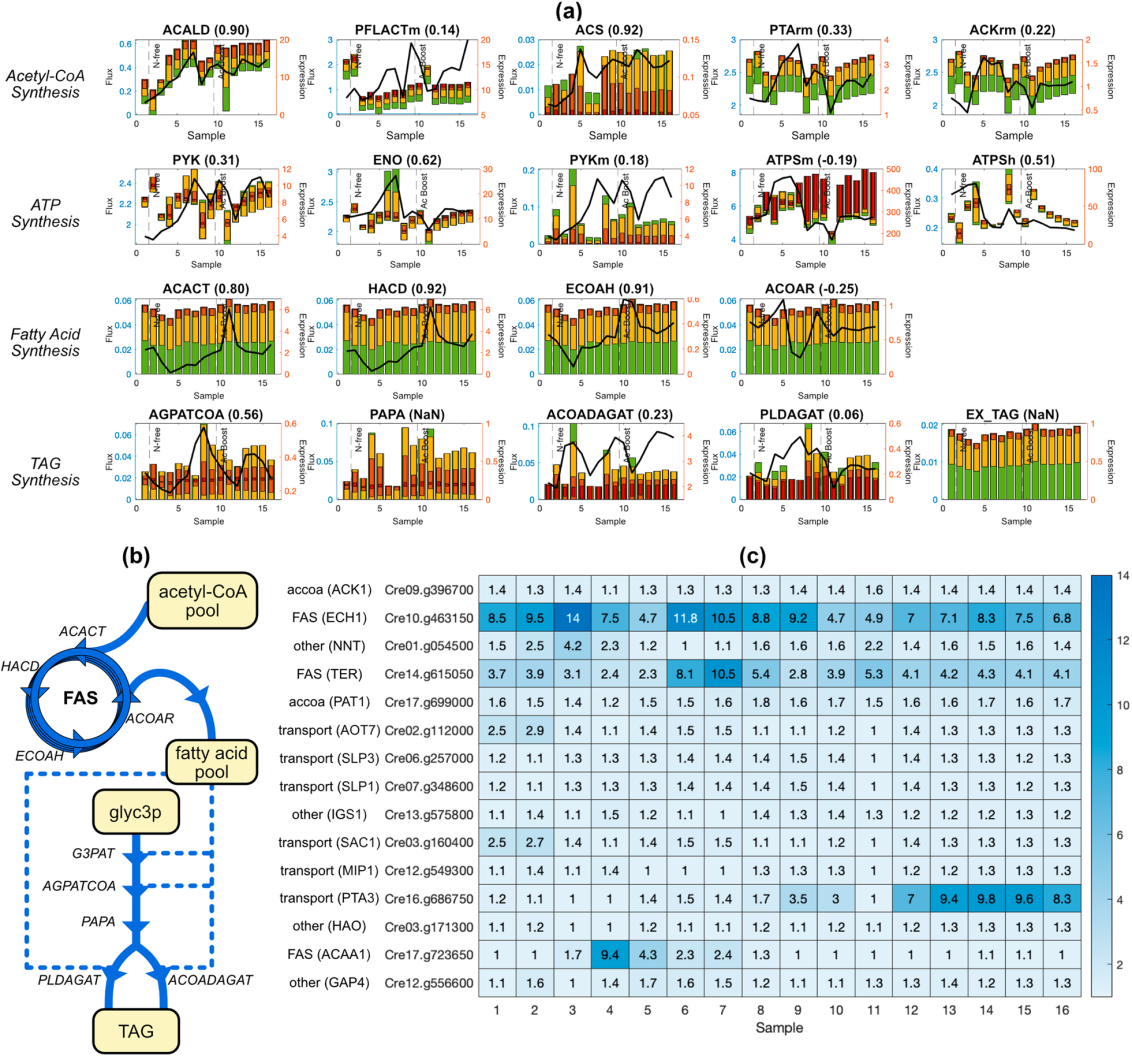
Various results of expression data interpretation through context-specific PC-model. **a** Is a selected collection of PC-FVA results across 16 samples regarding acetyl-CoA synthesis, ATP synthesis, *De novo* fatty acid synthesis (FAS), and TAG synthesis pathway reactions. The bar plot shows the variability of each metabolic reaction and is coupled to the left y-axis in mmol/gDW/h. Green, yellow, orange, and red bars reflect the flux variability at $0\%$, $50\%$, $90\%$, and $99\%$ of maximum TAG synthesis rate (i.e., EX_TAG flux), respectively. The black line plot shows the expression level of the reaction and is coupled to the right y-axis. In the case of isozymes presence, the black line plots the numerical sum of all isozyme levels. The calculated Spearman’s rank correlation ($\rho$) between the FVA result and expression is listed in the bracket. Vertical dashed lines divide time-series samples into pre-wash, N-free, and post-boost phases. **b** Demonstrates a simplified reaction network around FAS and the TAG synthesis pathway. Different fatty acid, their derivatives, and different TAG are not differentiated. **c** Shows the suggested overexpression level (folds) of the top 15 bottleneck proteins to maximize TAG production while minimizing deviation from the measured RNA-Seq (through the protein abundance constraints). Gene symbols and functional categories of each protein are shown to the left of each gene ID (row labels)

The main merit of the context-specific PC-model is converting expression data to metabolic fluxes, which are insightful both independently and comparatively across samples. For example, the maximum TAG production rate is slightly reduced by ammonium-free medium and slightly promoted by the acetate boost (Fig. 4a), yet it does not translate to the ‘actual’ accumulation rate, as any point on the bar is possible for *C. reinhardtii* to operate on.

Using the final PC-model, including calibrated $k_\text {eff}$, we performed a systems-level analysis of dynamic shifts in the proteome and fluxome for mixotrophic TAG production. Given the best-fit proteome for every RNA-Seq sample, we computed the corresponding fluxome using protein-constrained flux variability analysis (PC-FVA) with TAG production rate constrained to $\ge 0\%$, $50\%$, $90\%$, and $99\%$ of the maximum (Fig. 4a). For each reaction, we computed the Spearman rank correlation ($\rho$) between the max flux from PC-FVA and the total abundance of all transcripts associated with the reaction. From this procedure, among all 1876 enzymatic reactions, we classify 130 reactions as expression-dependent ($\rho \ge 0.8$), 218 reactions as expression-correlated ($0.5 \le \rho < 0.8$), and 1528 reactions as expression-independent ($\rho < 0.5$), while spontaneous reactions are always expression independent (Additional file 3: Fig. S3).

From these PC-FVA results, especially with high optimum percentages (orange and red bars in Fig. 4a), we find that the key reactions for TAG production can be categorized into acetyl-CoA synthesis, ATP synthesis, *De novo* synthesis of free fatty acids, and TAG synthesis (Fig. 4a).

##### Acetyl-CoA synthesis

The majority of acetyl-CoA was supplied from acetaldehyde dehydrogenase (ACALD), formate C-acetyltransferase (PFLACTm), and phosphotransacetylase (PTArm). Of these reactions, ACALD is the only expression-dependent (Spearman rank $\rho =0.9$) reaction (Fig. 4a). ACALD is associated solely with the gene Cre17.g746997 and shows high expression correlation even for PC-FVA computations with TAG production $\ge 99\%$ of the maximum (Fig. 4 red bars). TAG production is strongly dependent on ACALD flux, which in turn is strongly expression-dependent. Thus, Cre17.g746997 is an overexpression candidate to increase acetyl-CoA supply.

On the other hand, PFLACTm and PTArm fluxes are uncorrelated with gene expression (Spearman rank $\rho <0.5$). PFLACTm, producing acetyl-CoA by converting pyruvate to formate, is likely dictated by the upstream pyruvate mass balance. PTArm catalyzes the highest flux of acetyl-CoA production and does not appear to be dictated by either its own (Cre09.g396650 or Cre17.g699000) expression or acetate kinase (ACKrm) (Cre09.g396700 or Cre17.g709850) expression. Acetyl-CoA synthetase (ACS) produces acetyl-CoA using acetate like PTArm, and it also coincides with the expression. However, its flux is nearly 100-fold lower than PTArm. Furthermore, maximum TAG dilution is achieved when ACS flux is low, and PTArm flux is high (Fig. 4a). This result suggests that ACS flux can be controlled through gene expression and that to maximize TAG production, its expression should be repressed.

##### ATP synthesis

ATP production is dominated by cytosolic pyruvate kinase (PYK) and mitochondrion ATP synthase (ATPSm), while the chloroplast ATP synthase (ATPSh) activity is relatively low across all samples. This mode of ATP synthesis observed for TAG production contrasts sharply with PC-FBA simulations of optimal growth that showed large proteome allocation toward photosystems I and II (Fig. 2a). The acetate boost starts from sample 10—prior to the boost, ATPSh flux shows moderate variability and low flux relative to mitochondrial ATPS. After the acetate boost, ATPSh flux variability is even narrower, and TAG production is maximized with lowered ATPSh flux. The good correlation of ATPSh flux with gene expression (Spearman rank $\rho =0.51$) indicates that *C. reinhardtii* is programmed to reduce photosystem-based ATP synthesis under mixotrophic growth with nitrogen limitation. Indeed, the dynamic regulation of ATP production between mitochondria and chloroplast has been studied, but no transcription factor is found [34].

PYK produces ATP by converting phosphoenolpyruvate to pyruvate. PYK flux and protein allocation differ notably between the optimal mixotrophic and heterotrophic flux networks (2PG/PEP to PYR in the cytosol in Fig. 2A). During TAG production, PYK flux is not strongly correlated with gene expression, indicating that other constraints, such as mass balance, determine its flux. Specifically, enolase (ENO) produces phosphoenolpyruvate, which is the primary substrate of PYK. ENO is correlated with expression of Cre12.g513200 (Spearman rank $\rho =0.62$). In turn, PYK flux is highly correlated with ENO (Spearman rank $\rho =0.994$); therefore, both PYK and ENO flux can be controlled by regulating the Cre12.g513200 gene.

##### De novo fatty acid and TAG synthesis

An intuitive way to maximize TAG production would be to overexpress its direct biosynthesis genes. This strategy has been applied for multiple algae species, including *C. reinhardtii* [35]. TAG is synthesized from glycerol 3-phosphate by a sequence of five reactions: glycerol-3-phosphate (G3PAT), 1-hexadecanoyl-sn-glycerol 3-phosphate O-acyltransferase (AGPATCOA), phosphatidate phosphatase (PAPA), acyl-CoA diacylglycerol acyltransferase (ACOADAGAT), and phospholipid diacylglycerol acyltransferase (PLDAGAT). Three acyl groups are attached in the process, using acyltransferase reactions (G3PAT, AGPATCOA, ACOADAGAT, PLDAGAT). TAG accumulation has been increased by overexpressing AGPATCOA in *Cyanidioschyzon merolae* [36], PLDAGAT in *C. reinhardtii* [35] and *Phaeodactylum tricornutum* [37]. These strategies work by pulling carbon flux to TAG.

Our simulations are consistent with these observations in that maximum TAG accumulation requires elevated expression of the acyltransferase proteins. Namely, the minimum required flux of AGPATCOA was above $\sim 0.01$ to achieve max TAG flux (Fig. 4a—*TAG synthesis*). The two diacylglycerol acyltransferases (ACOADAGAT and PLDAGAT) are also required to maximize TAG flux, but because either reaction can be used, each flux has a minimum requirement of zero.

Our simulations indicate that another, possibly more critical, strategy for TAG production is to provide ample acyl-CoA by overexpressing free fatty acid synthesis cycle reactions: Acetyl-CoA C-acyltransferase (ACACT), 3-hydroxyacyl-CoA dehydrogenase (HACD), and enoyl-CoA hydratase (ECOAH), and trans-2-enoyl-CoA reductase (ACOAR). These reactions showed time-course flux patterns that were nearly identical to the TAG dilution reaction (EX_TAG) (Fig. 4a). Three of these reactions (ACACT, HACD, and ECOAH) are highly correlated with gene expression (Spearman rank $\rho =$ 0.8, 0.92, and 0.91). Therefore, overexpression of these genes would directly increase flux. Indeed, a sharp overexpression of these genes coincides well with the acetate boost (Fig. 4a, *De novo synthesis*). Finally, the ACOAR reaction is uncorrelated with gene expression (Spearman rank $\rho =-0.25$); however, due to mass balance constraints, its flux is entirely determined by the flux of the preceding three reactions in the cycle.

#### Engineering strategies for enhancing TAG productivity

Next, we developed a tool to find optimal, system-level debottlenecking strategies for metabolic engineering. This tool is formulated as a linear program (Methods): TAG production is maximized subject to all context-specific PC model constraints while also allowing for a user-defined total protein overexpression “budget” (*E*). The optimal solution to this problem provides the set of highest priority targets for protein overexpression. Using this tool, we identified protein overexpression strategies that were consistent with the transcriptome changes observed in the acetate-boosted experiment. For example, all three free fatty acid synthesis genes (ECH1, TER, ACAA1) that were highly correlated with reaction flux (ECOAH, HACD, ACACT) were identified as overexpression targets (Fig. 4b). ECH1 requires an average of 8.2-fold overexpression across 16 samples, the most out of all modelled genes.

Additionally, two of the acetyl-CoA synthesis genes (*ackA and PAT1*), corresponding to ACKrm and PTArm reactions, were identified as overexpression targets. Interestingly, the algorithm did not identify the gene for ACALD as an overexpression target, despite it being a key step in Acetyl-CoA synthesis. This result is consistent with the high expression levels of ACALD-associated transcripts (Fig. 4a, ACALD); therefore, no further debottlenecking is required. In fact, this result suggests that the expression levels for PATrm and ACKrm-associated genes may need to be increased further to achieve TAG production higher than that observed.

Finally, we identified additional overexpression candidates that may be candidates for future engineering. These proteins include six transporters, including those for sulphate (*SLP1, SLP3*), phosphate (*PTA3*), and amino acids (*AOT7*) (Fig. 4b). Other targets include proteins associated with amino acid biosynthesis (*IGS1*), glycolysis (*GAP4*), redox balance (*NNT*), and glyoxylate metabolism (*HAO*) (Fig. 4b).

Apart from debottlenecking TAG synthesis through overexpression, we can also block competing reactions by gene deletions to achieve the maximum potential. For example, multiple studies have shown that starchless mutants of *C. reinhardtii* exhibit significantly more TAG accumulation [38, 39]. Our flux simulations are consistent with these results, showing reduced TAG production capabilities when starch synthesis reaction is active (Additional file 4: Fig. S4, STARCH300S). Shin et al. discovered that knocking out phospholipase A2 (Cre02.g095000) would increase *C. reinhardtii* lipid productivity by up to $64 \%$ [40]. Our simulation also resembles that an active phospholipase A2 will always reduce TAG productivity by deviating diacylglycerol away from TAG synthesis (Additional file 4: Fig. S4, PLPSA2). Yunus et al. and Kato et al. showed that deleting the fatty acyl-ACP synthase would better preserve the system free fatty acid level, and therefore boost hydrocarbon accumulation in cyanobacteria [9, 41]. In *C. reinhardtii* simulation, we observed a different but similar phenomenon, wherein the reaction fatty acid CoA ligase hinders TAG production (Additional file 4: Fig. S4, FACOAL). Experimentally knocking out the three genes above forces their respective fluxes to zero, which are the flux values OVERLAY predicts to enable maximum TAG production.

Another possible TAG productivity-enhancing approach is to supplement the medium with additional carbon sources. The measured transcriptomes indicated the expression of transporters for alternative carbon substrate uptake, including l-glutamine, d-ribose, and d-lactate. By adding these carbon sources to the *in silico* growth media, we confirmed that the observed gene expression levels support the uptake of these carbon sources, albeit at slow rates (see Additional file 6: Table S3). However, our simulations indicate that supplementing these carbon sources would not boost TAG production under the current transcriptome because the added carbon can not alleviate the main bottleneck, fatty acid biosynthesis.

## Discussion

### *C. reinhardtii* as a synthetic biology chassis

Microalgae *C. reinhardtii* has been studied for decades as a cell factory, producing both bulk metabolites and, more recently, expressing heterologous genes for non-native value-added products. Although many established methods are available for bulk metabolite production, in some cases, they are still far from optimal productivity based on our simulation results. Indeed, the recent adaptive laboratory evolution of *C. reinhardtii* has increased both growth rate (by up to 300%) and product yields (DHA production by 90%) [42]. Using genome-scale modelling, especially with the context-specific PC-model pipeline, *C. reinhardtii* may become an economically-efficient cell factory for bulk chemicals after multiple iterations of optimization. On the other hand, the optimization for non-native products is more complicated, yet it can be highly impactful for the (bio)chemicals industry due to the potential for sustainable production of high-value products, especially when non-biological synthesis routes are unavailable.

Besides *C. reinhardtii* and algae in general, *Escherichia coli* has also been extensively studies as a synthetic cell chassis. The main advantages of *C. reinhardtii* over *E. coli* are its lower carbon emission and simplicity in cultivation, which are both more significant in bulk chemical productions setup. Therefore, biofuel and potential food production has been the main interest in *C. reinhardtii* cell factories. On the other hand, *E. coli* is the best studies microorganism with even more established knowledge and synthetic biology tools available to researchers. The production of certain value-added chemicals by *E. coli*, including various vitamins and nutraceuticals, has reached commercialization stage [43, 44]. We believe that OVERLAY, as a general computational tool, is also capable of helping to boost the productivity of *E. coli* cell factories.

### Evaluation and application of OVERLAY

Our PC-model formulation and OVERLAY pipeline provide several advantages over existing methods. First, our PC-model computes optimal fluxomes in response to changes in the allocation of the proteome. This proteome, in turn, is highly consistent with measured transcriptomes through a sequence of convex and nonconvex quadratic optimization problems. Second, to perform context-specific simulations, we do not require choosing an arbitrary gene expression threshold for turning on/off reactions based on transcript abundance—this has been a challenge in existing methods [12]. Third, our pipeline enables the identification of optimal overexpression targets. This method requires only one parameter to be adjusted: *E* (total protein overexpression budget), which can be determined using a simple procedure. Finally, our method enables using transcriptomics to quality-control genome-scale reconstructions and their annotations, which are found through persistent discrepancies between optimal protein and measured transcript abundances.

Given all the advantages offered, the application of OVERLAY can be extended from metabolic engineering to a broader scientific inquiries. Importantly, OVERLAYovercomes the shortcomings (optimality assumption and incomplete scope) of FBA and PC-FBA by imposing constraints and calibrating rate constants using transcriptomics measurements that are consistent with observed phenotypes. For example, mixotrophic regulations and metabolisms in algae are a research focus for scientists. A recent study by Vidotti et al. measured time-course transcriptomic and proteome data for autotrophic, mixotrophic, and heterotrophic *Chlorella vulgaris* [45]. These measurements can be easily incorporated onto the newest *C. vulgaris* metabolic reconstruction iCZ843 using OVERLAY for deeper metabolic insights [46]. We anticipate OVERLAY to consistently help to address scientific problems related to steady state and transition state metabolism. Additionally, because OVERLAY can find errors in knowledge-based M-models (i.e., Additional file 6: Table S2), it may help researchers to update their understanding, such as discovering new metabolic pathways or enzymatic functions. These utilities are not offered by the original M-model or even PC-model solely, as shown previously in optimal autotrophic and mixotrophic photosystem activity predictions.

In general, PC-model and OVERLAY is a simple yet effective tool for understanding and manipulating cellular metabolism through gene expression, making it potentially valuable for various applications. The prediction results can be used for practical decision-making in various research fields such as biotechnology, infectious disease, and cancer. We believe OVERLAY will benefit the system biology and metabolic engineering community.

### Prospect: incorporating other omics data

With more multi-omics data measurements in recent studies, we think it is worth mentioning the potential of OVERLAY to uniquely incorporate more omics data to achieve higher modelling performance.

#### Kinetome

Kinetome refers to the collection of cellular enzymatic rate constants [47]. Due to the scarcity of measured enzyme kinetic, OVERLAY is designed to be capable of conducting high-quality *C. reinhardtii* modelling without kinetome data. We believe OVERLAY can incorporate available kinetome data by explicitly setting and fixing individual rate constants in the PC-model before nonconvex QP. Available kinetome data would likely increase the modelling performance.

#### Proteome

Proteome profiling data, if available, is a substitute of transcriptomic data for OVERLAY. In this study, OVERLAY uses transcriptome data to approximate cellular proteome, which is potentially less accurate than using proteome data directly. However, RNAseq remains more accessible than proteome profiling, making it more practically useful in modelling for biotechnological applications. If proteome profiling is available but not for all proteins, it can still be useful in partially constraining metabolic flux or verifying its consistency with transcriptomic data.

#### Metabolome

Metabolomic data refers to the presence or concentration of intracellular metabolites. Recently, the GEM community has developed various protocols, such as MetaboTools and matTFA toolbox, to incorporate metabolomics data into M-model [48, 49]. These protocols can be adopted in conjunction with OVERLAY to improve model quality. These fall outside the scope of this study; however, they are promising directions for future work.

## Conclusion

In this work, we developed a computational pipeline OVERLAY for building a context-specific, protein-constrained genome-scale model (PC-model), starting from metabolic reconstruction and transcriptomics data. We showcase the utility of PC-model for deciphering how complex gene expression dynamics drive system-level fluxome shifts in *C. reinhardtii* using published time-course transcriptomics data. Using PC-FBA, we recapitulate metabolic hallmarks of autotrophic, heterotrophic, and mixotrophic growth. Importantly, the protein constraints are required to accurately simulate respiro-fermentation (overflow) metabolism. We then use time-course RNA-Seq data to investigate the over-production of triacylglycerol (TAG) in response to acetate supplementation under nitrogen limitation [29]. Our pipeline generated context-specific models for each experimental time point (over four days of culture), with very high consistency between 1495 modelled proteins and measured transcriptomes ($R^2$ between 0.95 to 0.963, median $R^2=0.958$). We then determined which metabolic fluxes were controlled by gene expression. By comparing simulated fluxes and measured transcriptomes across the 16 time-course RNA-Seq samples, we could categorize all gene-associated reactions into 130 expression-dependent (Spearman rank $\rho \ge 0.8$), 218 expression-related (Spearman rank $0.5 \le \rho < 0.8$, and 1528 expression-independent (Spearman rank $\rho < 0.5$) reactions.

To enable researchers to systematically identify optimal overexpression targets, we developed a novel optimization-based tool. Using the tool, we identified key gene expression bottlenecks for TAG overproduction. The tool recapitulated known bottlenecks (e.g., the acyltransferase steps in TAG biosynthesis). Furthermore, we identified several novel overexpression targets to improve TAG overproduction further, including genes encoding sulphate, phosphate, and amino acid transporters; glyoxylate metabolism, and redox balancing.

## Material and methods

### Merging iCre1355 and iGR774 metabolic model and curation

Instead of using *C. reinhardtii* M-model iCre1355 only, we decided to plug in a newer chloroplast M-model, which contains a more up-to-date understanding of chloroplast metabolism. We merged M-model iCre1355 (cellular model) and iGR774 (chloroplast model) by first deleting all chloroplast metabolites and reactions in the cellular model. The chloroplast model was slightly modified (see Additional file 7: Table S4), and its transported reactions were matched to the dead-end transportations in the cellular model (see Additional file 7: Table S5–S7). The newly merged M-model has 1354 genes, 2641 reactions, and 2240 metabolites. We curated the gene-reaction rules (stored in the model as model.rules field) using complex data from ChlamyCyc 8.0 [32]. Only enzyme complexes with multiple subunits were curated, but not any protein-monomer enzymes (Additional file 7: Table S8). The starch metabolism pathway and appropriate rules were also added to the merged model (Additional file 7: Table S4). We also opened the lower bound of reaction GAPDHi and GAPDH_nadp to allow reverse reactions (Additional file 7: Table S4). The script written to merge and modify M-models is MergedModel.m, which calls functions in COBRA Toolbox on MATLAB to load and manipulate M-models [11, 50]. We used the Kyoto Encyclopedia of Genes and Genomes (KEGG) and BiGG Models as general references in M-model modifications [51, 52].

### Obtaining and processing expression data for case study

Raw reads of RNA-seq data (E-GEOD-56505) for the TAG case study were downloaded as FASTQ files [29]. We downloaded the NCBI genome assembly GCF_000002595.2.gbff and parsed the GenBank file into a FASTA reference transcript [53]. Reads were aligned using Bowtie2 with default settings and quantified using Samtools and Salmon [54–56]. The complete quantified vector is denoted as $t_{cp} \in \textbf{R}^{17713}$, which is further parsed a modelled transcript vector $t \in \textbf{R}^{1495}$.

### Protein constraints implementation

Our PC-model formulation is shown below.

1 $$\begin{aligned}{} & {} \max _{v,p,x,e} c^Tv \end{aligned}$$

2 $$\begin{aligned}{} & {} s.t.\ Sv = 0 \end{aligned}$$

3 $$\begin{aligned}{} & {} v^\text {lb} \le v \le v^\text {ub} \end{aligned}$$

4 $$\begin{aligned}{} & {} Cx \le p \end{aligned}$$

5 $$\begin{aligned}{} & {} e_\text {for} + e_\text {rev} = B \cdot diag(r) \cdot x \end{aligned}$$

6 $$\begin{aligned}{} & {} -K_\text {eff}^\text {avg}Ie_\text {rev} \le v \le K_\text {eff}^\text {avg}Ie_\text {for} \end{aligned}$$

7 $$\begin{aligned}{} & {} 0 \le p \le p^\text {ub} \end{aligned}$$

8 $$\begin{aligned}{} & {} p^Td \le P, \end{aligned}$$

where $v\in \mathbb {R}^{2641}$, $p\in \mathbb {R}^{1495}$, $x\in \mathbb {R}^{1000}$, and $e_\text {for}, e_\text {rev}\in \mathbb {R}^{1876}$ denote metabolic flux, proteome concentration, complex concentration, and enzyme concentration, respectively.

By adopting this formulation, we assumed the following:

1. The total amount of metabolic proteome may not exceed a weight fraction of the dry weight, which is further referred to as the ‘proteome budget’ and denoted by a scalar (*P*) in mg/gDW.
2. Each annotated gene in the M-model is transcribed and translated to a unique protein whose molecular weight can be estimated by its protein sequence.
3. Rate constant of a certain enzyme is fixed regardless of reactions. This will greatly reduce the complexity of the problem, especially the nonconvex quadratic programming problem (nonconvex QP) in the later section.
4. Enzyme concentration upper bounds but not forces the respective reaction flux. Enzymes are currently not compartmentalized.

The protein constraints were implemented in the M-model by adding four sets of variables and four sets of constraints. Variables are defined as follows:

1. Protein dilution: protein concentrations (or abundances) in nmol/gDW. Proteins are uniquely defined for each gene in the M-model.
2. Complex formation: complex concentrations in nmol/gDW. We define ‘complex’ as a unique protein combination that can sufficiently catalyze any single reaction. The list of complexes is obtained by parsing rules in M-model (parseGeneRule.m).
3. Enzyme formation: enzyme concentrations in nmol/gDW. We define ‘enzyme’ as a collection of indifferent complexes that can catalyze a certain reaction. A pair of forwarding and reverse enzymes are added for each enzymatic reaction, and no enzyme is added for spontaneous reactions.
4. Enzyme dilution: One dilution reaction for each forward or reverse enzyme.

Extra constraints were added to the model as follows:

1. Each complex may not exceed the abundance of available protein subunit, according to *C* (4). *C* is a matrix containing complex subunit information. Excess proteins are allowed.
2. The sum of forward and reverse enzyme equals the total complex, according to *r* and *B* (5). *B* is Boolean matrix mapping complexes and enzymes and further enzymatic reactions. Vector *r* denotes the ratio between the rate constant of each complex and the average enzymatic rate constant, or $K_\text {eff}^i = r_i \cdot K_\text {eff}^\text {avg}$. We first estimated *r* as below (estimateKeffFromMW.m):
  9 $$\begin{aligned} r_i^\text {ori} = \left ( \frac{X_i}{\frac{1}{N} \sum ^N_{i=1}X_i} \right ) ^{3/4}, \quad X = C^{-1}d, \end{aligned}$$
  which is scaled according to the enzymatic surface area as other studies [30, 31].
3. Enzymatic reaction fluxes are restrained by respective forward and reverse enzyme levels through the average rate constant of $65s^{-1}$.
4. Protein concentrations are collectively constrained by the proteome budget *P*, according to protein molecular weight vector *d* in mg/nmol. We assumed *P* being a constant across all growth conditions, and the weight fraction of total proteome to be $600mg/g \text {DW}$. Modelled proteome weight fraction within the total proteome ($\% modelled$) can be estimated using the complete transcript $t_{cp}$ and modelled transcript *t*, as well as molecular mass vector for complete transcript $d_{cp}$ and for modelled transcript *d*. Thus, *P* is approximated as below.
  10 $$\begin{aligned} P \approx 600 \cdot \% modelled = 600\cdot \left ( \frac{ t^Td}{t_{cp}d_{cp}} \cdot \frac{\text {length}(t)}{\text {length(t)}-\text {length}(\text {NaN} \; t)} \right ) , \end{aligned}$$
  where $\text {NaN} \; t$ denotes the collection of modelled transcripts of which are not present in $t_{cp}$. This may happen either for genes in mitochondria and chloroplast genome, or due to the presence of genes in the M-model whose identifiers do not map to any genes in the transcriptomics data. The estimated *P* are shown by Additional file 5: Fig. S5, and we chose $P = 150$ for this dataset.

We collected a complete protein sequence FASTA file using NCBI genome assembly *Chlamydomonas reinhardtii* v5.5 (GCF_000002595.2.gbff), *Chlamydomonas reinhardtii* chloroplast reference genome (NC_005353.1), and *Chlamydomonas reinhardtii* mitochondrial reference genome (NC_001638.1) [53]. This FASTA was constructed by extracting all locus tags and respective protein sequences into a plain text file (fastaParsing.m). It was used to calculate the molecular mass of modelled proteins (calcProteinMM.m). The PC-model construction processes above are also automated in a MATLAB file as pcModel.m. The solving time of PC-FBA is around 0.3 seconds on our device, which is six times more than its respective FBA.

### Overlaying processed RNA-Seq data onto PC-model using convex QP

We proposed a methodology to interpret the underlying cellular metabolism for a given RNA-seq data using the PC-model (overlayMultiomicsData.m). Assuming the proteome vector is similar to the mRNA vector, we formulated a quadratic objective function, subject to constraints (2)–(8):

11 $$\begin{aligned} \min _{v,p,x,e} (diag(w)(p-t))^T (p-t), \end{aligned}$$

where *t* denotes the modelled transcript abundance vector, and *w* is a weighting vector for each transcript. This finds the proteome vector closest to the measured transcriptome while maintaining underlying metabolic feasibility. We defined

12 $$\begin{aligned} w_j = \Biggl \{ \begin{array}{ll} \frac{1}{t_j}, &{} t_j > 0 \\ 1, &{} t_j = 0 \\ 0, &{} \text {NaN} \; t_j, \end{array} \end{aligned}$$

which increases the weighting of lowly transcribed and un-transcribed genes. This is essential to keep the unexpressed proteins absent from the context-specific model, although other weighting functions might be feasible too. The expression measurement was unavailable for some modelled proteins ($\text {NaN} \; t_j$), in which case the weighting was assigned to zero. *t* was scaled to satisfy

13 $$\begin{aligned} t^Td = P, \end{aligned}$$

which put *t* and *p* into the same magnitude. This guaranteed the possibility that objective function (11) might reach zero value from solving.

This is a convex QP problem and can be solved using commercial LP solvers such as Gurobi Optimizer or IBM ILOG CPLEX [57, 58]. The optimization took around five seconds for both solvers. Defining the best-fitting protein vector is solved to be $p'$, and we replaced constraint (7) with constraint (14) for the base PC-model to make it sample-specific.

14 $$\begin{aligned} p_j \Biggl \{ \begin{matrix} 0 \le p_j \le p_j^\text {ub}, &{} \text {NaN} \; t_j \\ (1-s)p_j' \le p_j \le p_j', &{} otherwise. \end{matrix} \end{aligned}$$

We added a slack term ($s=0.02$) into constraint (13) for two practical reasons: leaving the proteome budget for unmeasured transcripts in the unbiased analysis and making the de-bottlenecking algorithm easier to implement. We found that eliminating the slack also indirectly restricts unmeasured proteins due to proteome budget depletion, which would impact the downstream analysis. In our practice, adjusting *s* within a reasonable range would not significantly affect the result.

### Estimating system level enzymatic rate constants using nonconvex QP

Although it is impractical to experimentally measure all enzymatic rate constants, they can be systematically estimated using this PC-model formulation. Given the *Q* set of samples of the same strain under different conditions, we rewrote objective function (11) into (15). The intuition was to find the single vector *r* that allows the best-fitting result for all RNA-seq samples:

15 $$\begin{aligned} \min _{v^k,p^k,x^k,e^k,r} \sum ^Q_{k=1} (diag(w')(p^k - t^k))^T (p^k-t^k), \end{aligned}$$

subject to constraints (2)–(8) for each of the *Q* samples. For example, constraint (2) effectively becomes

16 $$\begin{aligned} \begin{pmatrix} S_1 &{} \cdots &{} 0 \\ \vdots &{} \ddots &{} \vdots \\ 0 &{} \cdots &{} S_Q \end{pmatrix} \begin{pmatrix} v_1 \\ \vdots \\ v_Q \end{pmatrix} = 0. \end{aligned}$$

We also simplified the weighting by decorrelating $w'$ with relative abundance, greatly speeding up the computation:

17 $$\begin{aligned} w_j' = \Bigg \{ \begin{matrix} 0, &{} \text {NaN} \; t_j \\ 1, &{} otherwise. \end{matrix} \end{aligned}$$

Vector *r* was made a variable with the following constraints:

18 $$\begin{aligned}{} & {} \frac{1}{N} \sum ^N_{i=1} r_i = 1, \end{aligned}$$

19 $$\begin{aligned}{} & {} r_i \Bigg \{ \begin{matrix} 0.1r^\text {ori}_i \le r_i \le 1.9r^\text {ori}_i, &{} \exists t^k_j \ge t^k_\text {avg}, &{} C_{j,i}>0 \\ r_i = r^\text {ori}_i, &{} \forall t^k_j < t^k_\text {avg}, &{} C_{j,i}>0. \end{matrix} \end{aligned}$$

The constraints above formulate a nonconvex QP that is *Q* times the size of the convex QP. By constraint (19), we preserved the original $r_i$ with low subunit abundance, which prevented the solver from prematurely modifying the rate constant. This is inferring that a more comprehensive rate constant estimation can be achieved by including data from various metabolic modes while stacking up data from similar metabolic modes will benefit little; on the other hand, adding each set of data exponentially increases the computational cost. Thus, we first performed hierarchical clustering by MATLAB Statistics and Machine Learning Toolbox to categorize 16 RNA-seq samples into four groups (Additional file 2: Fig S2), which were then used group averages as ‘samples’ to estimate *r* using nonconvex QP [59]. Procedures above can be done by overlayMultiomicsData.m with ‘keffEstimate’ option set to true.

The QP was solved by Gurobi Optimizer version 9.1.2, a state-of-the-art LP solver that supports nonconvex bilinear optimizations [57]. The optimization took around 2000 seconds on a laptop with an Apple M1 chip and 16 GB of memory, although the computation time can vary widely for the same problem size with different data samples.

### De-bottlenecking and unbiased network analysis

Acknowledging errors and uncertainties in the data and workflow, we applied a de-bottlenecking optimization onto data-specific PC-models to mitigate the effect of a few bottlenecking proteins without shifting the landscape (proteinDebottleneck.m). This was done by adding a variable term to the constraint (14), which becomes

20 $$p_i \Bigg \{ \begin{array}{ll} 0 \le p_i \le p^\text {ub}_i, &{} \text {NaN} \; t_i \\ (1-s)p_i' \le p_i \le p_i' + \epsilon _i, &{} otherwise, \end{array}$$

where $\epsilon$ is a variable vector with an assigned error budget *E* (also referred to as overexpression budget):

21 $$\begin{aligned} \sum \epsilon \le E, \end{aligned}$$

while other constraints are the same as the data-specific PC-model, optimized to the objective function (1) using the FBA algorithm, referred to as protein-constrained flux balance analysis or PC-FBA. We conducted this LP by varying error budget values and eventually chose $E=20$, where the curve of optimal objective values versus *E* reached a constant slope (see Additional file 7: Table S9). This means no single protein is blocking the objective function, and therefore it was a suitable state for the downstream analysis. The result can also be interpreted as a suggested list of proteins to overexpress. The optimization took roughly 1.0 s using Gurobi Optimizer.

To further understand the metabolic capabilities under each expression data, we used PC-FVA for an unbiased analysis. For each data-specific model, FVA of all metabolic reactions *v* was done to find $v_{min}$ and $v_{max}$ at the optimal percentages of 0%, 50%, 90%, and 99%.

## Supplementary information

**Additional file 1: Fig. S1.** The complete record of consistency of simulated proteomes to transcriptomics before and after nonconvex QP by OVERLAY. This is an extended version of Fig. 3ab.

**Additional file 2: Fig. S2**. Hierarchical clustering result of 16 time-course RNA-seq sample.

**Additional file 3: Fig. S3**. Histogram of Spearman’s ranking coefficient for all metabolic reactions. This supplements Fig. 4, where all Spearman’s coefficients are calculated.

**Additional file 4: Fig. S4**. PC-FVA prediction results for starch synthesis reaction, phospholipase A2 reaction, and fatty acid CoA ligase reaction. This figure can be interpreted using the caption of Fig. 4a.

**Additional file 5: Fig. S5**. Bar plot of proteome budget estimation using dataset.

**Additional file 6**: Tables S1 to S3 for results section.

**Additional file 7**: Tables S4 to S9 for methods section.

## Data Availability

OVERLAY is available to download at https://github.com/QCSB/OVERLAY-Toolbox. The MATLAB code and pre-computed workspace for the work are available at https://github.com/QCSB/algal-pcFBA.
