# Supplementary figures and images for: Novel context-specific genome-scale modelling explores the potential of triacylglycerol production by Chlamydomonas reinhardtii
Source: Microb Cell Fact. 2023 Jan 17;22:13. doi: 10.1186/s12934-022-02004-y (PMC9847032; doi:10.1186/s12934-022-02004-y)

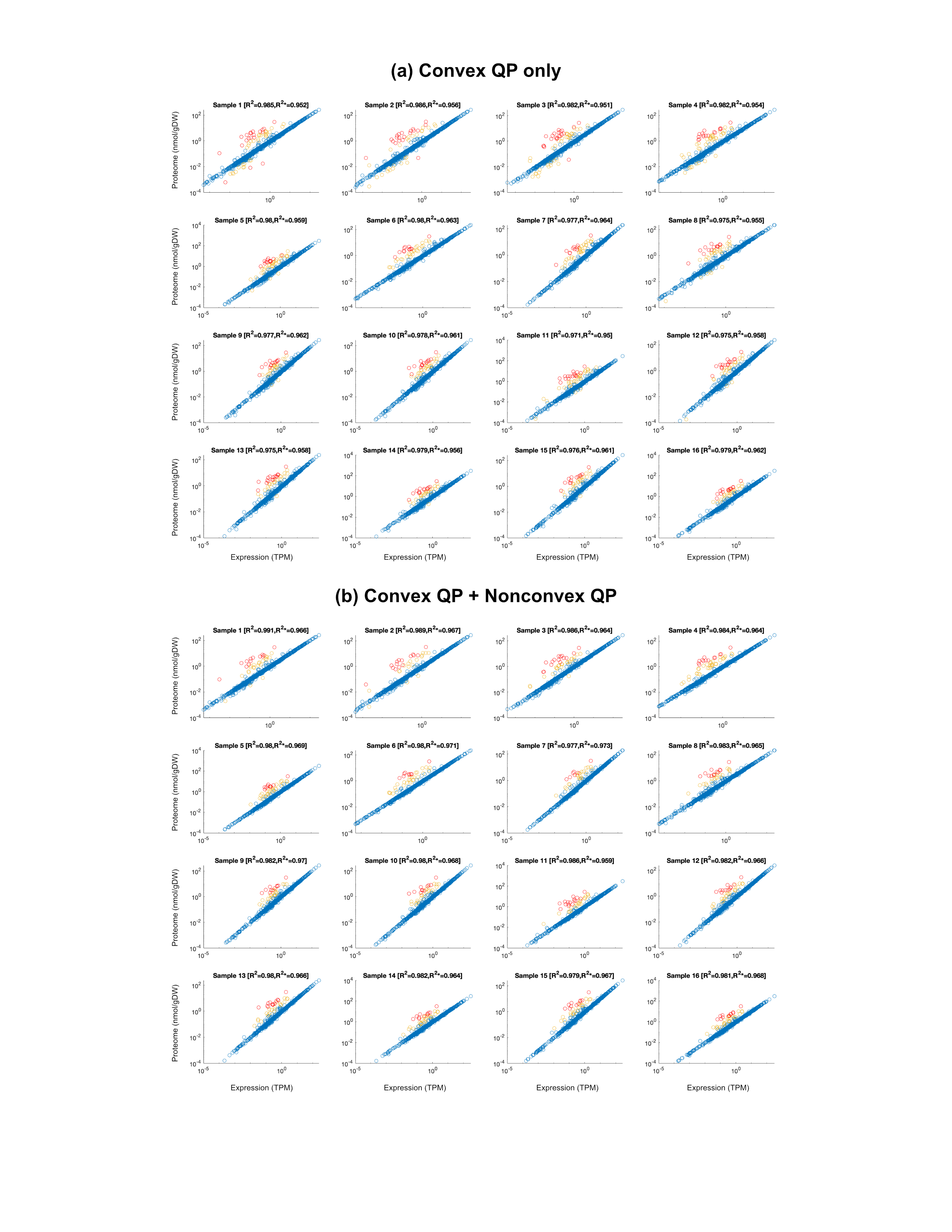

Supplement: Supplementary file 1 — Additional file 1: Fig. S1. The complete record of consistency of simulated proteomes to transcriptomics before and after nonconvex QP by OVERLAY. This is an extended version of Fig. 3ab. [file 12934_2022_2004_MOESM1_ESM.png]

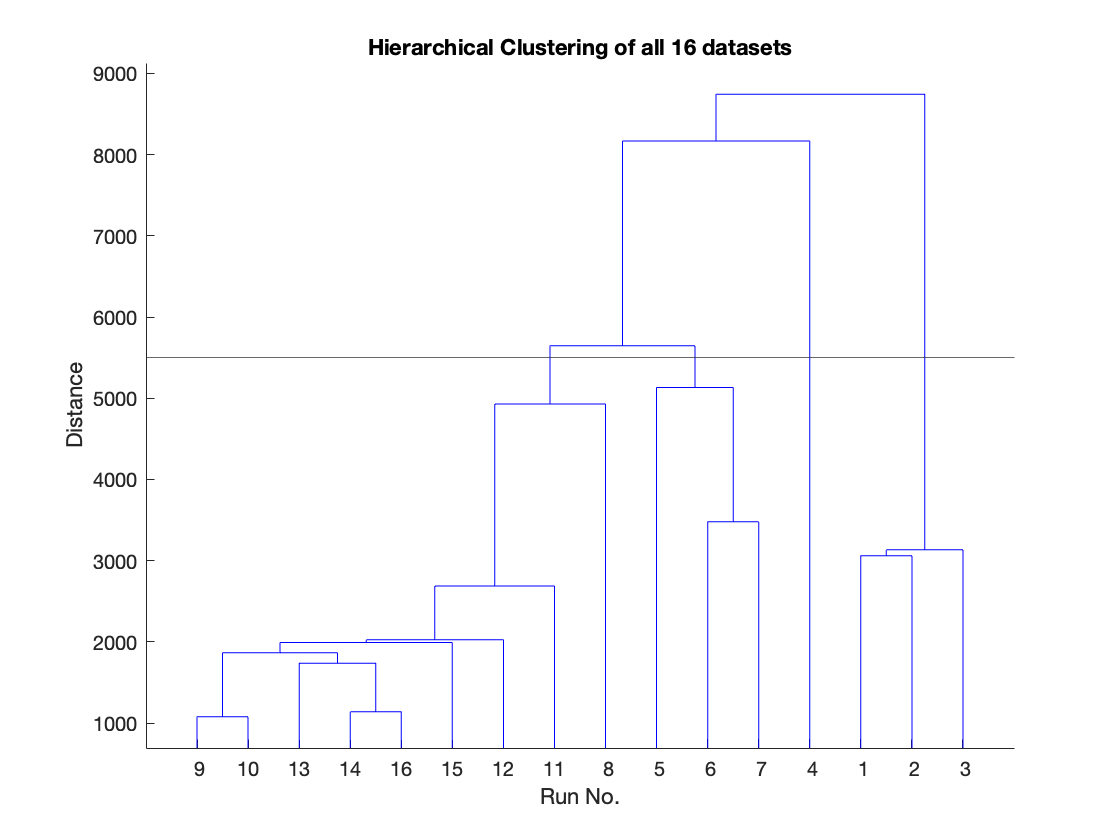

Supplement: Supplementary file 2 — Additional file 2: Fig. S2. Hierarchical clustering result of 16 time-course RNA-seq sample. [file 12934_2022_2004_MOESM2_ESM.png]

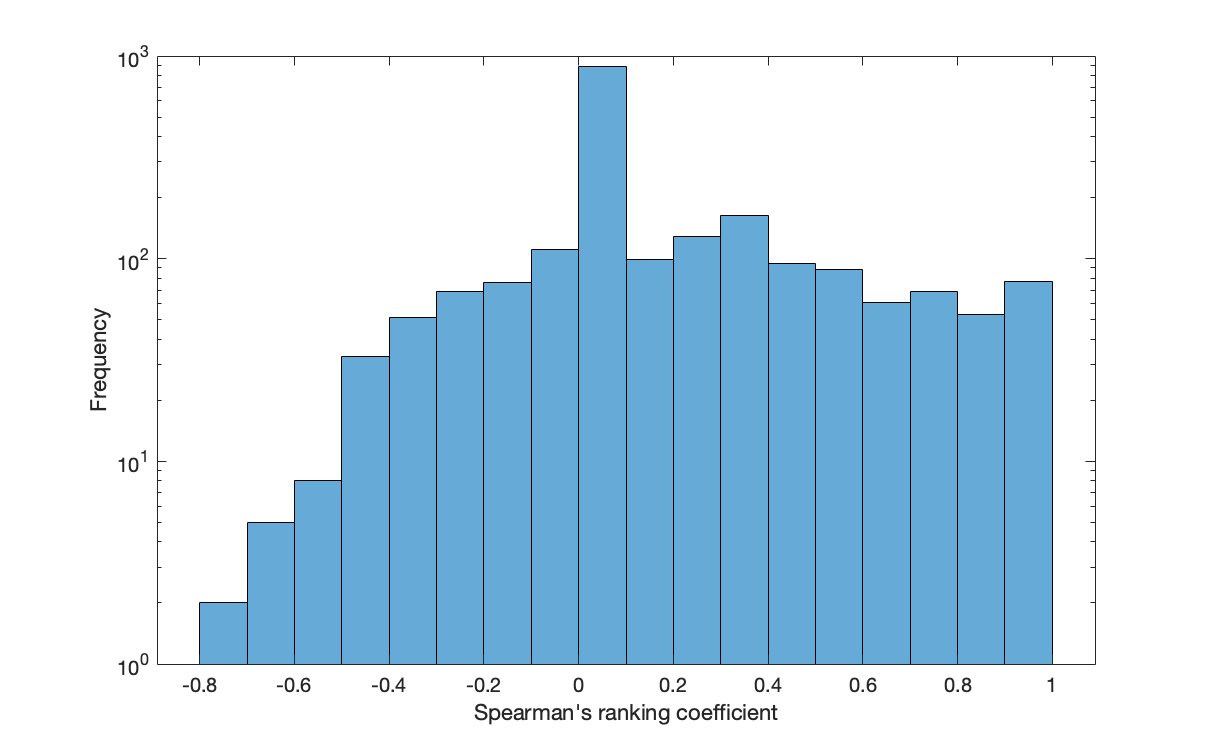

Supplement: Supplementary file 3 — Additional file 3: Fig. S3. Histogram of Spearman’s ranking coefficient for all metabolic reactions. This supplements Fig. 4, where all Spearman’s coefficients are calculated. [file 12934_2022_2004_MOESM3_ESM.png]

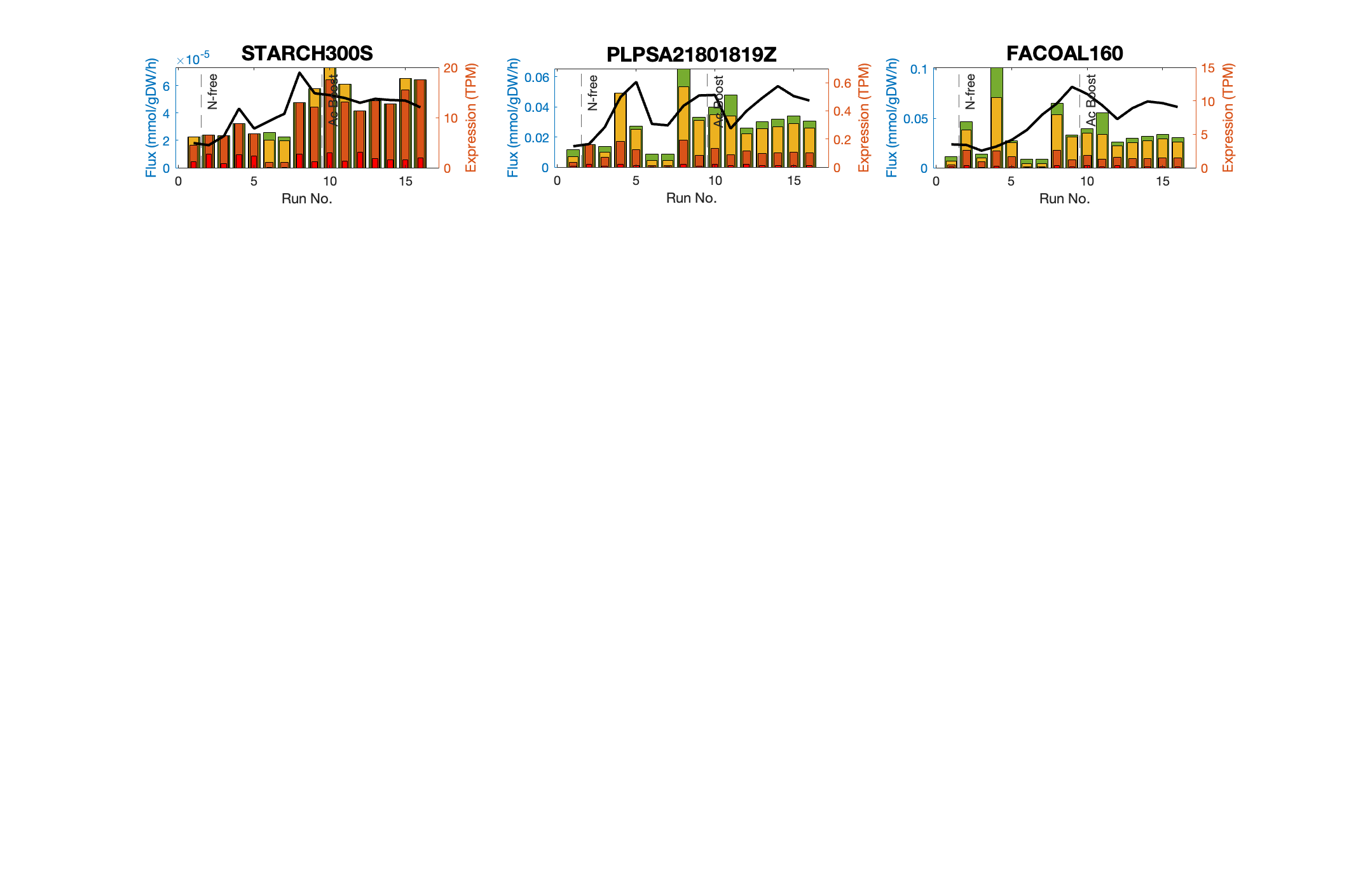

Supplement: Supplementary file 4 — Additional file 4: Fig. S4. PC-FVA prediction results for starch synthesis reaction, phospholipase A2 reaction, and fatty acid CoA ligase reaction. This figure can be interpreted using the caption of Fig. 4a. [file 12934_2022_2004_MOESM4_ESM.png]

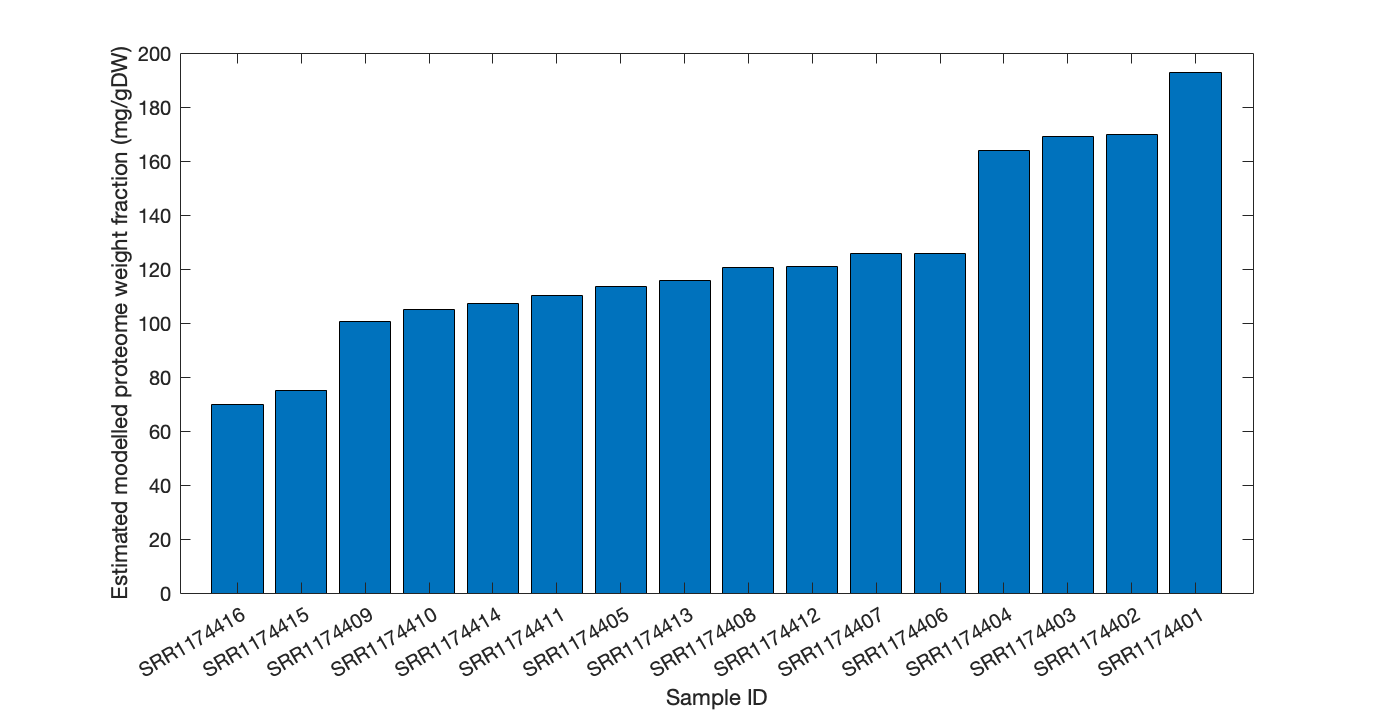

Supplement: Supplementary file 5 — Additional file 5: Fig. S5. Bar plot of proteome budget estimation using dataset. [file 12934_2022_2004_MOESM5_ESM.png]
